# Molecular causality in the advent of foundation models

**DOI:** 10.1038/s44320-024-00041-w

**Published:** 2024-06-18

**Authors:** Sebastian Lobentanzer, Pablo Rodriguez-Mier, Stefan Bauer, Julio Saez-Rodriguez

**Affiliations:** 1 Heidelberg University, Faculty of Medicine and Heidelberg University Hospital, Institute for Computational Biomedicine, Heidelberg, Germany; 2 Helmholtz AI and TU Munich, Munich, Germany

**Keywords:** Systems Biology, Causality, Foundation Models, Inductive Bias, Latent Spaces, Computational Biology

## Abstract

Correlation is not causation: this simple and uncontroversial statement has far-reaching implications. Defining and applying causality in biomedical research has posed significant challenges to the scientific community. In this perspective, we attempt to connect the partly disparate fields of systems biology, causal reasoning, and machine learning to inform future approaches in the field of systems biology and molecular medicine.

## Abstract (web summary)

Correlation is not causation: defining and applying causality in biomedical research has posed challenges. This perspective attempts to connect the partly disparate fields of systems biology, causal reasoning and machine learning, to inform future approaches in systems biology and molecular medicine.

## Graphical abstract

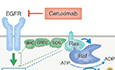

## Introduction

Correlation is not causation. As simple as this widely agreed-upon statement may seem, scientifically defining causality and using it to drive our modern biomedical research is immensely challenging. Since its first description by Aristotle approximately 2500 years ago (Aristotle and Owen, 2016), causal reasoning (CR) remained virtually unchanged until it experienced significant formal and mathematical advancements (Pearl, 2009b; Angrist et al, 1996; Card and Krueger, 2016) and a recent resurgence in the field of machine learning (Kaddour et al, 2022; Tejada-Lapuerta et al, 2023; Chernozhukov et al, 2024). In parallel, biomedicine has made major leaps in the past century, in particular in the context of the development of high-throughput and large-scale methods.

In the field of systems biology, great hopes of deriving causal insights from large-scale omics studies have largely been thwarted by the complexity of molecular mechanisms and the inability of existing methods to distinguish between correlation and causation (The 1000 Genomes Project Consortium, 2010; Glocker et al, 2021; Listgarten, 2023). In part, this may be caused by the divergence between two general approaches to systems biology: bottom-up and top-down modelling. The bottom-up approach uses detailed mechanistic models that are built from the ground up, and as such shows parallels to CR. The top-down approach, on the other hand, is characterised by the use of large-scale data-driven models, and as such shows parallels to machine learning.

In medicine, randomised clinical trials show that, in a lower-dimensional context, we can reliably identify causal effects. By controlling “all” relevant covariates in a trial (via the principle of the gold-standard, randomised, double-blind, and placebo-controlled trial), we isolate the causal effect of the controlled variable, i.e., the treatment. In the language of Pearl’s Do-Calculus (Pearl, 2012), we measure the outcome of, for instance, *do ("Treat with Vemurafenib")* when conducting a clinical trial on V600E-positive melanoma (Chapman et al, 2011). However, translating this mode of reasoning into the high-dimensional space of modern omics poses enormous challenges. The dramatically larger parameter space of models at the molecular level leads to problems in method performance and result identifiability (Squires and Uhler, 2022; Esser-Skala and Fortelny, 2023; Chis et al, 2011), as well as in model explainability (Carloni et al, 2023). In this perspective, we discuss the current connections between CR and molecular systems biology in the context of these challenges. We will elaborate on three main points:

- Biases and what they mean for CR, particularly in the context of biomedical data;
- The role of prior knowledge (PK) in CR and how to translate PK into suitable biases;
- The role of foundation models in molecular systems biology and their relationship to CR.

## Background

### Causal discovery and inference

The field of CR distinguishes between **causal discovery**—the process of building causal hypotheses from data—and **causal inference**—the process of predicting specific outcomes when given data and the causal relationships known a priori about the system.

Causal discovery is more expensive than inference both computationally and data-wise, because it involves distinguishing between correlation and causation and extracting generalisable relationships from the data (Heinze-Deml et al, 2018; Squires and Uhler, 2022). For modern systems biology, this means that methods for causal discovery typically require large amounts of experiments. Highly parameterised models such as neural networks increase this requirement even further. As such, many consider causal discovery in molecular biomedicine a scaling problem (Willig et al, 2022; Branwen, 2020).

**Figure 1:**
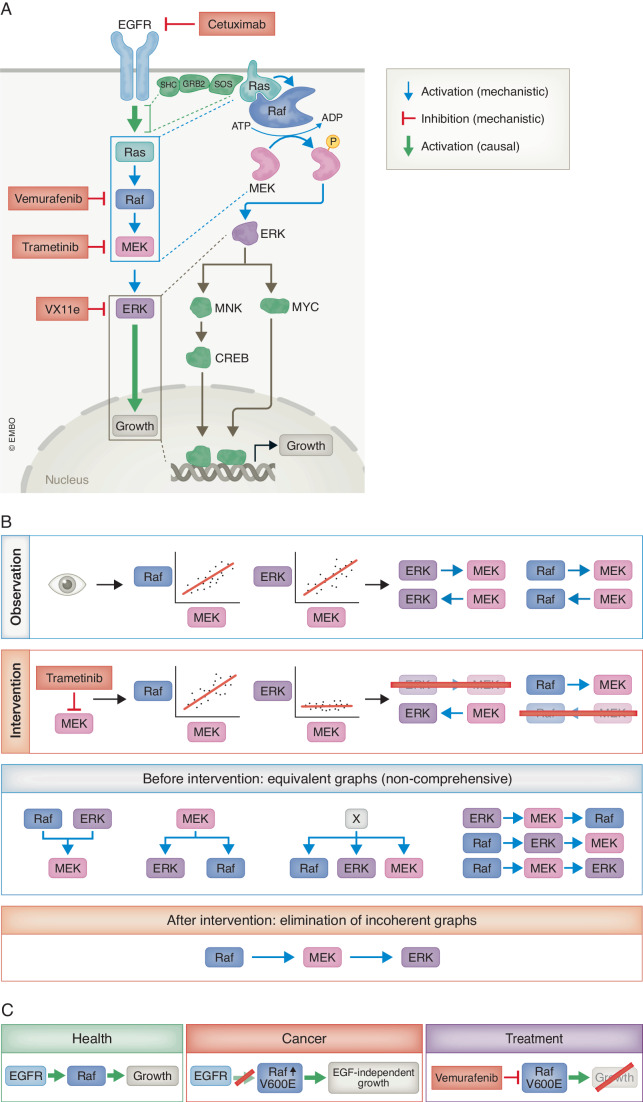
The EGFR-ERK pathway. (**A**) The EGFR, upon activation, leads to growth via a linear cascade of activations. Displayed are several direct causal (mechanistic) interactions, such as activation via protein-protein interactions (blue) and inhibition by drugs (red); and two indirect causal interactions (green), which occur via molecular intermediates. (**B**) Blue boxes: Observational correlations between protein activities of components of the pathway do not allow concrete conclusions regarding the exact causal structure of the pathway, leading to a class of equivalent explanations for the observations (not all are shown). Orange boxes: Upon intervention, we can exclude certain possibilities, closing in on the true structure of the causal graph. (**C**) The clinical implications of causal reasoning in molecular systems biology. The independent activation of Raf via the V600E mutation leads to cancerous growth, which can be treated by inhibiting the overactive Raf protein. Mechanistic explanations are often not required in the presence of causal knowledge. MEK mitogen-activated protein kinase kinase, ERK extracellular signal-regulated kinase, EGFR epidermal growth factor receptor.

Causal inference, on the other hand, focuses on quantifying the causal effects of one variable on another within the framework of already hypothesised causal relationships. This approach leverages PK about the assumed causal links, which in the causal field are often encoded using directed graphs. Most inference mechanisms perform better when including PK at some point in the process, as has been observed in biomedical research (Hill et al, 2016). This allows researchers to represent both the causal connections between variables and their directionality, which is required to understand how changes in one variable might lead to changes in another. For instance, in the case of the EGFR-ERK signalling pathway, a graph would depict Raf activation leading to MEK activation, which in turn leads to ERK activation (Fig. 1A). This clear representation of directionality is important for causal inference, as it ensures that analyses focus on the effect of upstream changes on downstream outcomes. For example, when analysing phosphoproteomic data to assess the impact of inhibiting MEK, a graph-based approach would guide researchers to correctly attribute subsequent changes in ERK to this specific intervention (Fig. 1B). Without this causal framework, one might mistakenly interpret correlations as bidirectional influences or overlook confounding factors, leading to incorrect conclusions (Fig. 1C). However, the inference is also very sensitive to the completeness of the PK that is applied, and most biomedical PK is far from complete (Garrido‐Rodriguez et al, 2022). For instance, the function of more than 95% of all the known phosphorylation events that occur in human cells is currently unknown (Needham et al, 2019; Ochoa et al, 2019). In contrast to causal discovery, scaling plays a smaller role in causal inference. Here, the main problems are incompleteness and identifying the “right” biases to apply.

### The ladder of causality

Orthogonally to the distinction between causal discovery and inference, we can also distinguish between different levels of causality. Pearl’s ladder of causality roughly distinguishes three types of CR in increasing order of power: observation, intervention, and counterfactuals (Pearl and Mackenzie, 2018). While the inferences we wish to make in biomedical research are often of the counterfactual type (e.g., “would Raf inhibition lead to a decrease in ERK activation if the media contained Epidermal Growth Factor?”), the data we have available are typically observational (e.g., “the levels of Raf and MEK activity are correlated”) and sometimes interventional (e.g., “targeting Raf with CRISPR leads to a decrease in ERK activity”). Generating interventional or even counterfactual inferences from observational data is a major challenge, if not impossible, depending on the characteristics of the system under study (Pearl, 2009a).

There are approaches to delineate interventional inference from observational data, such as the “natural experiments” framework (Angrist et al, 1996; Card and Krueger, 2016). However, these approaches are by nature even more data-hungry than using interventional data, as they often do not use the full breadth of the dataset (Imbens and Lemieux, 2008). Therefore, in biomedical research, there has been a push towards generating large-scale interventional data, for instance by performing CRISPR/Cas9 screens with single-cell resolution (Dixit et al, 2016). Current developments of CR in the biomedical field thus mostly focus on these types of data.

### Deduction and induction

In CR, we can also distinguish between deductive and inductive reasoning. Deductive reasoning is the process of deriving a conclusion from a set of fixed and known premises. “All men are mortal, Socrates is a man, therefore Socrates is mortal” is a classic example of deductive reasoning. In biomedical research, this is typically the process of deriving a conclusion from a set of PK. For instance, having PK of the linear activation cascade (Fig. 1A), and that Vemurafenib will inhibit Raf activity, allows us to deduce that giving Vemurafenib will inhibit growth of cancer cells (Fig. 1C) (Chapman et al, 2011).

Inductive reasoning, on the other hand, involves making generalisations from specific observations. Testing the hypothesis above, we apply Vemurafenib in a clinical trial of V600E-positive melanoma and find that it is clinically efficacious (Chapman et al, 2011). Commonly, we then use induction to infer from this limited cohort that the treatment may be effective in the entire population. We could further infer that Vemurafenib may be an effective remedy in other V600E-positive cancers as well, or that inhibiting this cascade may be a general mechanism of action of anti-cancer agents in cancers that display ERK pathway overactivation (Bollag et al, 2012). In the molecular realm, we could further infer that the inhibition of other components of the cascade, such as EGFR or MEK, may also be promising target leads (Savoia et al, 2019).

The main difference between deduction and induction is that the former is logically complete—i.e., if the premises are true and the argument is valid, the conclusion must also be true. However, deduction is also more limited in scope than induction. In biomedical research, we often have to rely on inductive reasoning because we cannot feasibly test all hypotheses in a deductive manner. As a result, the *inductive biases* we introduce into our models (i.e., those mechanisms in the model that help with inductive reasoning) are a pivotal part of performing CR in biomedical research.

## Bias

### Meaning and examples of biases

Biases are systematic prejudices of a model towards certain outcomes. Humans make frequent use of biases so that they can function in a complex world with limited cognitive resources (Gopnik et al, 2004). In fact, we often presume causality from observation (i.e., we “jump to conclusions”), which is indicative of a strong inductive bias (Tenenbaum et al, 2011). A good *heuristic* is the application of a suitable bias to a problem, such that the solution can be considered acceptable despite limited resources.

In machine learning, we can distinguish between useful and harmful biases. Harmful biases are common issues in the technical process of training models; they include, for instance, sampling bias, selection bias, and confirmation bias (Mehrabi et al, 2019; Squires and Uhler, 2022). While addressing harmful biases is a crucial part of machine learning, we will not discuss them further in this perspective.

Useful biases, on the other hand, are biases that are introduced into a model to improve its performance. Since most models developed in biomedical research and the broader machine learning community are inductive models, one of the most discussed useful biases is *inductive bias* (Baxter, 2000). For instance, PK on protein interactions can impact inference on activation cascades; only upstream proteins can activate downstream proteins, not vice versa.

### Why do we need biases?

Humans will be the gold standard for common-sense reasoning for the foreseeable future. However, human reasoning is limited by our sensory and mnemonic capacity; we cannot reason about high-dimensional data since we can neither perceive it nor keep it in memory. Machine learning offers a promising solution for addressing these complexities. However, the “No Free Lunch” theorems present a fundamental challenge: no single learning algorithm may be universally superior across all problem domains (Wolpert and Macready, 1995). Although they have recently been challenged (Goldblum et al, 2023), these theorems highlight the inherent difficulty in designing algorithms that generalise well from specific training data to new, unseen data. Inductive biases guide algorithms in making educated guesses about unseen data, thereby improving their generalisation capabilities (Goyal and Bengio, 2022).

This need for inductive biases is particularly apparent in the realm of biomedicine (Sapoval et al, 2022). Biomedical research operates within a framework constrained by limited and often high-dimensional data, stemming from the high costs of experiments, the scarcity of samples, and the inherent complexity of biological systems. Coupled with the natural variability of biological measurements, these factors result in a low signal-to-noise ratio, making it challenging to discern meaningful patterns. Inductive biases direct the learning process towards more relevant solutions by incorporating assumptions that enable more effective learning and interpretation, ensuring that models are not just statistically sound but also biologically meaningful.

Some central questions then arise:

- How explicit should we be in introducing biases, i.e., should the model determine its own biases, or do we force them on the model?
- How do we choose the right biases to introduce?
- How do we evaluate the biases we introduce?

## Bias from prior knowledge

The first question alone is highly debated in the wider field of machine learning and is related to the concept of the bias-variance tradeoff. The frequently quoted “Bitter Lesson” posits that we should refrain from inducing all but the most basic biases in our models, and that we should not view metrics as the ultimate measure of performance, but rather whether the model gets us closer to some truth (Sutton, 2019). However, it has been argued that many improvements that led to the models of today, such as convolution or attention, disprove this theory (Vaswani et al, 2017), and that the intrinsic complexity of real-world systems does not obviate, but rather necessitate, the integration of human insight into our learning frameworks (Brooks, 2019; Whiteson, 2019).

In systems biology, specifically, there is much interest in finding models with suitable biases to deal with constraints specific to the field, such as data availability and the incompleteness of PK (Locatello et al, 2018; Scholkopf et al, 2021; Aliee et al, 2021; Listgarten, 2023; Goyal and Bengio, 2022). Considering these constraints, the question is not whether to include PK in our reasoning, but which knowledge, when, and how (Whiteson, 2019).

### Prior knowledge

PK refers to information or data that is available to inform a learning process, enhancing the performance of the trained models and their ability to generalise. It can be used to inform the inductive biases of a model, either explicitly through the design choices and assumptions embedded into the models, or implicitly through the data and methods used in training. For this to be possible, biomedical entities and relationships must be clearly defined and represented unambiguously. Additionally, the diversity in our tasks and knowledge sources requires a flexible representation. Knowledge representation frameworks can aid in this process (Lobentanzer et al, 2023a).

In the biomedical field, there is a rich tradition of documenting biological knowledge at various levels of detail and focusing on different aspects of biology. Detailed mechanistic models provide mathematical descriptions of the dynamic interactions at a molecular, cellular, or organismal scale. Genome-scale networks, including metabolic and gene regulatory networks, offer comprehensive views of metabolic processes and gene interactions (Le Novère, 2015). Protein–protein interaction databases recapitulate either causal or non-causal interactions between proteins (Le Novère, 2015).

### Modelling on prior knowledge

The integration of PK into models is a non-trivial but essential process for moving from correlation to causation. PK can be used to derive inductive biases either *explicitly* or *implicitly*.

The explicit case typically involves a mathematical framework where a set of assumptions is explicitly stated and integrated into the model. Ordinary Differential Equation (ODE) models, logic-based models, rule-based models, and constraint-based models (Bordbar et al, 2014), all of which are commonly used in systems biology, explicitly incorporate different types of PK, can be fitted to data, and then be used to answer different types of causal questions. In the field of CR, Structural Causal Models can be used when mechanisms are unknown (Tejada-Lapuerta et al, 2023; Squires and Uhler, 2022). Their advantage is high efficiency in the face of scarce data, but they are highly reliant on the quality and comprehensiveness of the underlying PK (Gilpin, 2023).

In contrast, implicit integration of PK in models involves learning useful representations directly from the data, without the explicit inclusion of biological assumptions or causal knowledge. Learning mechanisms introduced as implicit biases can be simple (e.g., sparsity) or elaborate. Simple implicit biases include regularisation techniques that help models generalise by preventing overfitting (Tibshirani, 1996), or decisions about the types of prior distributions in Bayesian models (Risso et al, 2018). More elaborate are neural networks which employ specific architectural designs, such as Convolutional Neural Networks (CNNs) (LeCun et al, 1989), Recurrent Neural Networks (RNNs) (Hochreiter and Schmidhuber, 1997), or Transformers (Vaswani et al, 2017). Their advantages and disadvantages are inverse to those of explicit models (Gilpin, 2023), and their performance relies on the quality of collected data and the suitability of the experimental design.

As a result, choosing the best way to derive inductive biases from PK is not straightforward. Models that explicitly incorporate PK are more interpretable and can generalise effectively even when data are scarce (Gilpin, 2023). However, they are constrained by the accuracy of the existing knowledge and often struggle to scale to larger datasets (Kaplan et al, 2020; Ghosh et al, 2022). Models with implicit biases, on the other hand, particularly those typically found in deep learning architectures, excel at learning from large, high-dimensional datasets and offer flexibility across diverse domains. Yet, they suffer from limited interpretability, are prone to overfitting, and typically do not generalise well to scenarios not encountered during training, such as predicting the effects of new drugs or drug combinations, largely due to their lack of causal knowledge.

Hybrid models make a tradeoff between those extremes, which is why they have been found to be useful in systems biology, where data are currently scarce (AlQuraishi and Sorger, 2021; Nilsson et al, 2022; Faure et al, 2023; Roohani et al, 2023; Fortelny and Bock, 2020; Lotfollahi et al, 2023; Yuan et al, 2021). While some methods base their architecture on PK, others employ two learners side-by-side; one which is driven by explicit biases from PK, and one which learns from data. Frequently, these learners are also coupled in an end-to-end learning process, i.e., they “learn together.” This mode of learning aims to benefit from the “bias-free” nature of neural networks while simultaneously improving model performance in the face of scarce data via the added explicit bias.

## Causality in foundation models

There has been an enormous spike of interest in attention-based neural network models, in large part due to the success of Large Language Models (LLMs). While the high performance of LLMs is based on myriad technical improvements, the introduction of attention as an architectural bias has been a major contributor to their success (Vaswani et al, 2017). This has inspired the development of attention-based molecular models, most commonly for gene expression (Avsec et al, 2021; Theodoris et al, 2023; Cui et al, 2023). Some of these models are encoder-based, following the BERT architecture (Devlin et al, 2018), while others are decoder-based, following the GPT architecture (Brown et al, 2020). Encoder-based models are designed to learn embeddings from the pre-training process, which can be used to, for example, classify or cluster cells. Decoder-based models, in contrast, are generative and can be used to predict gene expression profiles directly. In both encoder- and decoder-based models, attention as a learning mechanism enables the integration of non-local information in a flexible manner. In a molecular model that reasons about gene expression, attention allows the integration of distant regulatory elements (Theodoris et al, 2023). However, this mechanism comes with a computational cost that increases exponentially with respect to the length of the input sequence (Han et al, 2023).

The generalist capabilities of LLMs have led to the designation of “foundation models” (Stanford CRFM, 2021). Foundation models are models that achieve high performance by training a generic architecture on extremely large amounts of data in a self-supervised manner. They can be fine-tuned for more specific tasks, because they are thought to derive generalisable representations and mechanisms by training on an amount of data large enough to learn the complexity of real-world systems. However, recent molecular foundation model benchmarks highlight clear discrepancies between the “foundational” aspirations of the pre-trained models and the real-world evaluation of their performance (Kedzierska et al, 2023; Boiarsky et al, 2023). Briefly, the benchmarks found that, on single-cell classification tasks, the proposed foundation models did not outperform simple baselines consistently when applied “zero-shot,” i.e., without fine-tuning. State-of-the-art methods such as scVI (Lopez et al, 2018) and even the mere selection of highly variable genes was often statistically indistinguishable from the highly parameterised methods, and sometimes even yielded better classification outcomes. However, these are early models, and it could still be argued that, in line with the scaling hypothesis, models may improve via a combination of the right architecture with sufficient amounts of data (Roth et al, 2024).

Indeed, molecular foundation models lag behind in size: while current-generation LLMs have around 100 billion parameters or more and are trained on enormous text corpuses (hundreds of billions to trillions of tokens), molecular foundation models have tens of millions of parameters (scGPT: 53 M, Geneformer: 10 M) and are trained on corpuses of tens of millions of cells, which (optimistically) yields hundreds of billions of individual data points. Thus, LLMs are currently about 2000 times larger than molecular foundation models, while arguably also dealing with a less complicated system. The question whether scaling will lead to the emergence of “foundational behaviour” in molecular models is still a matter of much debate (Schaeffer et al, 2023).

### Attention—and large amounts of data—is all you need?

Given enough data to train on - and ample funds for compute - is attention “all you need” to induce reliable biases in your model? While there are doubts regarding the reasoning capabilities of LLMs, GPT arguably “understands” language very well already, to the point where it can flawlessly communicate and synthesise information (Biever, 2023). This is what the term “foundation model” implies: the model has derived a generalisable representation of language, a tool that can be fine-tuned for a variety of language-related tasks. This behaviour is not possible without assuming some form of causality, even if it is not explicitly encoded in the model (Willig et al, 2022; Nichani et al, 2024).

In this light, what are the reasons to be sceptical about the capacity of molecular foundation models to understand the “grammar” of the cell?

#### Explainability

For one, large transformer models (i.e., billions of parameters) are not explainable due to their high complexity. As such, there is often no way to scrutinise their reasoning beyond the output they produce (Bommasani et al, 2021; Ennab and Mcheick, 2022). What seems simple in the case of language models—the famous Turing test can be performed by any human with a basic understanding of language—is exceedingly difficult in the molecular space, where many causal relationships are still unknown (Biever, 2023). Yet the only way to scrutinise and subsequently improve the reasoning capabilities of a model is precisely this explicit validation of its predictions in an interpretable setting.

While the creation of explicit molecular models (e.g., logic, structural causal, or ODE-based models) and the self-supervised training of molecular foundation models are methodologically very different, both can provide a hypothesis on causal structure that can be formulated as a network. Theodoris et al explore the attention layers of their Geneformer foundation model to explain the model’s reasoning (Theodoris et al, 2023). While some layers show clear patterns of attention, such as attending to highly connected or highly expressed genes, other layers are not as readily interpretable, much less so than explicit molecular models. Improving the explainability of methods regardless of their underlying mathematical formalisms will likely also increase our understanding of the biological processes that drive their predictions.

#### Benchmarking

Whether these complex layers reflect the true complexity of the underlying biology or are rather evidence for overfitting to the training data is not clear. One argument in favour of overfitting is the poor generalisation of the model in independent benchmarks (Kedzierska et al, 2023; Boiarsky et al, 2023). To determine whether molecular foundation models indeed capture generalisable causal representations of biology, dedicated benchmarks are needed. If possible, these should be run in an unbiased and crowdsourced manner (Saez-Rodriguez et al, 2016; Chevalley et al, 2022).

#### Causal bias

The GPT architecture that led to the recent breakthrough in LLM capabilities employs “causal self-attention,” describing an implicit architectural bias that prevents the model from “looking into the future”: for predicting the next token, only the previous tokens in the sentence can be used (Han et al, 2023). This leverages the implicit causality present in language, which incidentally is similar to one of the earliest formal descriptions of causality (in 1748), that “the effect has regularly followed the cause in the past” (Hume and Millican, 2007). Compared to language, the data that form the input of molecular foundation models do not implicitly contain causal information. The individual cells are in general not on a known trajectory, and the genes that are masked as part of the training objective are masked at random (Theodoris et al, 2023) or according to their information value (Cui et al, 2023), not because they are downstream (in some form) of the genes used for prediction. This fundamental difference between language and molecular models has so far not been explored theoretically or empirically.

### Causal latent spaces

Due to the fundamental limitation of human perception, dimensionality reduction is a popular workflow for data interpretation, typically via methods such as PCA, t-SNE, or UMAP (Nanga et al, 2021). The hope is that exploration and explanation in the lower-dimensional embedding space may be less challenging than in the original data, which assumes that the most important aspects of variability in the original data are captured in the reduced dimensions (Dyer and Kording, 2023). However, without explicit supervision, which is uncommon in biomedical datasets, the resulting latent spaces are rarely interpretable, and do not lend themselves to causal interpretation. In addition, they often suffer from biases that result from technical rather than biological factors (Chari and Pachter, 2023). In consequence, biological insight during the exploration of these latent spaces is often challenging due to the dominance of biases over the biological generative mechanism.

Performing causal inference in latent spaces could potentially solve some of these issues, but this requires that the latent space can be meaningfully navigated. “Moving through the latent space” reduces the number of variables that change upon intervention, making exploration simpler in theory. In practice, however, ease and sensibility of exploration depend completely on whether the inductive biases in the embedding process capture the underlying biology. In addition, latent spaces have no trivial connection to the real-world measurements they are based on. Each model instance generates its own, independent latent space; in consequence, the exploration of latent spaces is challenging and time-consuming.

Even if a given latent space can be explored, there is often no guarantee that interpolation between sensible latent representations also leads to sensible results. As an example, consider a prevailing issue of visual generative models in drawing human hands: images of hands typically involve mangled anatomy and an incorrect number of digits (Chayka, 2023). Even though there is a section in the latent space that represents hands, this does not represent the concept of a hand, but rather is guided by learning on many diverse pictures of hands. A section of this latent space may represent only a finger, and carry some information that next to a finger there usually is another finger. However, when generating the image, there is no mechanism to keep track of how many digits to add to any generated hand, leading to wrong anatomy.

**Figure 2:**
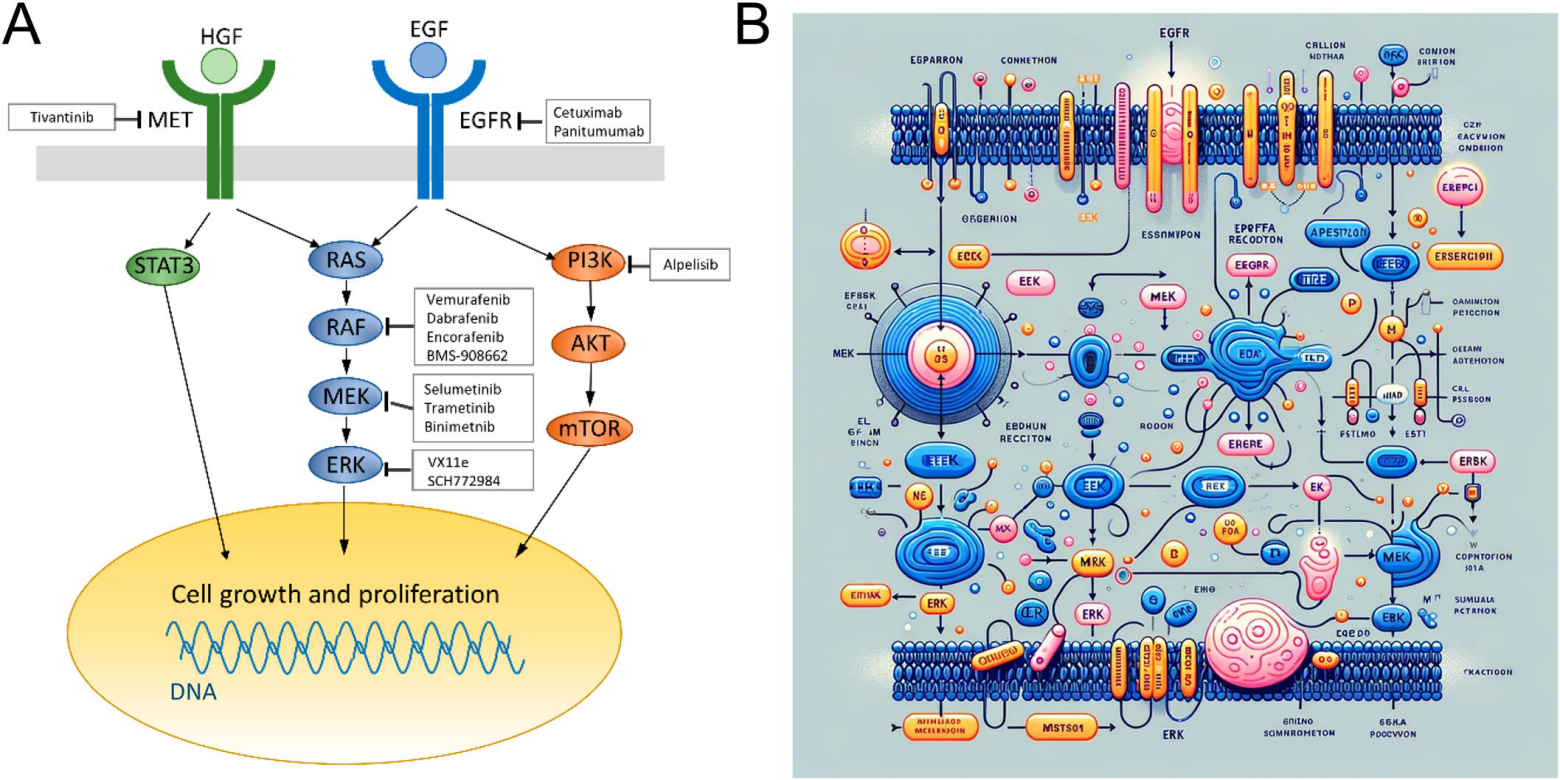
Pathway confabulation by generative AI model. (**A**) Figure of the EGFR-ERK pathway from (Miyamoto et al, 2017) (licensed under CC BY-SA 4.0). (**B**) Figure generated by OpenAI generative AI (ChatGPT 4 and DALL-E 3) upon request to “draw a minimalistic 2D schema of the EGFR pathway for growth involving MEK and ERK” (paraphrased).

Similarly, when exploring the latent space of a model of molecular signalling, there may be no guarantees that the model respects the concept of a given pathway when generating the signalling molecules involved. For instance, compare the human-made diagram of the EGFR-ERK pathway (Fig. 2A) to the one made by generative AI (Fig. 2B). While it is obvious that the DALL-E model simply retrieves nonsensical information from its latent space to synthesise a visually plausible image, it is not obvious how the transition from the clear and correct human visualisation to enabling foundation models to do the same should proceed. Of note, GPT-4 has excellent knowledge on all components of the EGFR-ERK pathway (Appendix Note), but still fails to instruct DALL-E to generate a sensible image.

If mastered, exploring and performing interventions in latent spaces promises many benefits: better generalisation and improved sample efficiency (Scholkopf et al, 2021), predicting the outcomes of interventions not observed at training time (Saengkyongam et al, 2023), or insights into the effect of different inductive biases in the model (Xia et al, 2021). However, to achieve this, it is essential to gain a better understanding of the properties of the learned embeddings and variables, for instance by performing “imagined interventions” in the latent space (Leeb et al, 2021) or by using model uncertainty for guiding the optimisation process in the latent space (Notin et al, 2021). Of note, many of the proposed solutions for more explainable latent spaces depend on architectures that may scale significantly worse than transformers (Kaplan et al, 2020; Ghosh et al, 2022).

## Conclusions

The debate between adopting scaling strategies versus the injection of biases from prior knowledge highlights a fundamental tension in modern biomedical research. The “Bitter Lesson” suggests a preference for general-purpose learning algorithms that implicitly learn biases from data. However, complex models often pose significant computational challenges (Squires and Uhler, 2022; Chevalley et al, 2022). Conversely, explicitly injecting biases from PK can lead to more specialised and efficient models that can generalise using relatively little training data, but may not scale. Hybrid models represent a promising middle ground. Researchers often rely on intuition to determine which biases to inject and, while no single model may universally excel (reflecting the “No Free Lunch” theorems), the blend of generalisation through scaling and specialisation through bias injection might provide a robust framework.

Theoretical work emphasises the need for interventions in causal discovery but does not yet address the influence of inductive biases (Eberhardt et al, 2012). The number of required interventions might be reduced significantly when complemented with high-quality observational data and appropriate biases, as suggested by neural causal models (Ke et al, 2019). Foundation models have embraced causal self-attention as a step towards integrating causality, but this alone may be insufficient.

In terms of data, large-scale collection is vital. Observational data are more readily available, but interventional data provide clearer causal pathways and can greatly enhance the model’s understanding of underlying biological processes (Lyle et al, 2023; Tigas et al, 2022). While the inclusion of a temporal axis can improve the amenability of observational data to causal inference, incorporating both observational and interventional data, coupled with mechanisms for deciding the right number and type of interventions, might improve model robustness and interpretability. The complexity and high cost of collecting good-quality data requires an efficient experimental design to maximise causal discovery with limited resources.

Foundation models challenge the “No Free Lunch” theorems by suggesting that certain architectural biases, learned from vast amounts of data, can yield generalisable and high-performing models (Goldblum et al, 2023). These biases, and how to transfer them from LLMs to systems biology, necessitate careful evaluation. As the biomedical field looks to these models for answers, it becomes crucial to develop frameworks that facilitate rapid development and exploration of ideas (Lobentanzer et al, 2023a; Lobentanzer et al, 2023b; Chevalley et al, 2022). A crucial aspect of these frameworks will be establishing benchmarks in the face of missing biological ground truth.

Systems biology has historically followed both knowledge-driven (bottom-up) and data-driven (top-down) approaches. Bottom-up systems biology, aiming to understand specific molecular mechanisms driving biological phenomena, has *de facto* been implementing CR, despite the two fields being largely disconnected. Meanwhile, top-down systems biology, inspired more by machine learning principles, has struggled with moving from correlation to causality. The methods and models described here offer the potential to converge these complementary approaches and scale our understanding to larger, more complex systems. However, it remains to be seen whether the future of biological modelling will be dominated by generalist models trained on vast datasets or by more nuanced, bias-inclusive architectures, informed by deep domain knowledge and specific data types (observational or interventional). We should explore these possibilities, balancing the drive for large-scale data with the need for precision and specificity, to realise the full potential of modern systems biology.

### Supplementary information

Appendix

## Glossary

**Attention (deep learning)**: A mechanism in deep learning that allows the model to focus on specific parts of the input data. Attention mechanisms are often used in natural language processing to focus on specific words in a sentence but can also be used in other domains.
**Bias (machine learning)**: Bias can be understood in two ways in the context of machine learning. (1) The first definition, and the one predominantly used in this article, is also referred to as statistical bias; a technical term referring to the assumptions made by a model to make predictions. This bias is a necessary part of any machine learning model. A model with high bias (low variance) pays very little attention to the training data and oversimplifies the model, which can lead to underfitting. This means it does not capture the complexity of the data and fails to learn the underlying patterns effectively. Conversely, a model with low bias (high variance) makes complex assumptions to fit the data closely, which can lead to overfitting, where the model captures noise in the data as if it were a true pattern. See also the bias-variance tradeoff. (2) The second definition is also known as algorithmic bias, and refers to the systematic and repeatable errors in a model due to faulty assumptions or data. It often reflects existing biases in the real world that the training data are derived from, but can also result from architectural choices in the model. As such, algorithmic bias can result from any stage in model training, from data collection to model deployment.
**Bias-variance tradeoff**: The concept in machine learning that bias and variance of a model are inversely related. The term implies that an optimal model finds a balance between bias (impact of the model on predictions) and variance (impact of the data on predictions). This balance depends on the complexity of the model and data.
Deductive vs. Inductive Reasoning: Deductive reasoning involves drawing specific conclusions from general statements or premises, whereas inductive reasoning involves making broad generalisations from specific observations. Deductive reasoning is often seen as more logically sound but less informative about the real world, while inductive reasoning is more exploratory but can lead to less certain conclusions.
**Do-Calculus**: Developed by Judea Pearl, Do-Calculus is a formal mathematical framework used in causal inference. It provides a set of rules for calculating the effects of interventions in probabilistic models, allowing researchers to infer causality from observational data.
**Foundation model**: A model that is trained on a large amount of data and can be used as a starting point for further model development (also referred to as fine-tuning). Foundation models are assumed to have learned generalisable patterns from their input data. To achieve this, they require large amounts of data and computing power.
Large Language Models: Large Language Models are advanced AI models trained on extensive text data. They are capable of understanding and generating human-like text, making them useful in various applications like translation, summarisation, and conversation. LLMs leverage vast amounts of training data to grasp nuances of language, context, and even some elements of human communication. They are the first commercially successful examples of foundation models.
**‘No Free Lunch’ Theorems**: These theorems in optimisation and machine learning suggest that no single algorithm is best for every problem. The performance of an algorithm is contingent on the specificities of the task and data at hand. This highlights the importance of choosing or designing algorithms that are well-suited to the particular characteristics of the problem being addressed. Related to the bias-variance tradeoff, partly opposed to the scaling hypothesis and foundation models.
**Overfitting**: A technical term referring to a model that captures noise in the data as if it were a true pattern. Overfitting tends to lead to high performance on the training data but poor performance on the test data. If a model has overfitted also to the test data, it will also perform poorly on new data, i.e., it will not generalise well.
**Prior knowledge**: A term referring to information that is available to inform a learning process. Often, this is the result of previous research.
**Randomised Clinical Trials**: Randomised clinical trials are experiments designed to test the efficacy of medical interventions. Participants are randomly assigned to groups receiving different treatments, including a control group, which typically receives a placebo or gold-standard treatment. To further minimise confounding factors, participants and administering doctors are often blinded to the treatment given. This method is considered the gold standard in clinical research for its ability to minimise bias and establish causality between a treatment and its outcomes.
**Scaling hypothesis**: The scaling hypothesis posits that the performance of a model increases with the amount of data it is trained on. Recently, it has come to describe the idea that, given enough data, complex model behaviours can emerge. The enormous success of current Large Language Models has been attributed to scaling, with the emergence of human-like language capabilities around the time of GPT-3. The ability to scale depends on several factors: the availability of data, parallelisation of training, adequate compute power with a parallel architecture, and a model architecture that can digest large amounts of data effectively.
**Self-supervised learning**: A type of machine learning where the model learns from the data itself, without the need for human labelling. This is achieved by training the model to predict certain properties of the data, such as the next word in a sentence, or the next frame in a video. Self-supervised learning is often used in the pre-training of foundation models. It typically requires a specific mechanism in the model to account for the lack of labelled data, such as the masking applied in the training of Large Language Models.
**Structural Causal Models (SCMs)**: SCMs are a type of statistical model used to represent and analyse causal relationships. They consist of variables and equations that describe how these variables interact causally. SCMs are particularly useful in causal inference as they allow for the analysis of how changes in one variable may cause changes in another.
**Underfitting**: A technical term referring to a model that does not capture the complexity of the data. Underfitting tends to lead to poor performance on both the training and test data.
**Variance (machine learning)**: A technical term referring to the sensitivity of a model to the training data. Describes how much the predictions of a model vary given different training data. High variance (low bias) in a model can lead to overfitting and thus harm generalisation. Conversely, low variance (high bias) can lead to underfitting and thus to a model that does not capture the complexity of the data. See also the bias-variance tradeoff.
