# Supplementary material for: Molecular causality in the advent of foundation models
Source: Mol Syst Biol. 2024 Jun 18;20(8):848–58. doi: 10.1038/s44320-024-00041-w (PMC11297329; doi:10.1038/s44320-024-00041-w)
Supplement: Supplementary file 1 — Appendix [file 44320_2024_41_MOESM1_ESM.pdf]

Appendix Note

The following is a reproduction of a conversation with ChatGPT 4 on Feb 21, 2024. The original can be found at this link: https://chat.openai.com/share/06b55b22-d514-452d-a5fd-f7bcecfd0563

User question

What are the components of the EGFR - ERK pathway, and how are they connected?

ChatGPT 4 answer

The Epidermal Growth Factor Receptor (EGFR) - Extracellular Signal-Regulated Kinase (ERK) pathway is a key signaling cascade involved in regulating cellular processes such as proliferation, differentiation, and survival. This pathway is part of the larger family of Mitogen-Activated Protein Kinase (MAPK) pathways, which play critical roles in transmitting signals from the cell surface to the nucleus. The EGFR-ERK pathway specifically involves the activation of the EGFR, a receptor tyrosine kinase, leading to a series of downstream events that culminate in the activation of ERK, a MAPK. Here's an overview of the components and their connections:

1. **EGFR Activation**: The pathway is initiated when a ligand, such as Epidermal Growth Factor (EGF) or Transforming Growth Factor-alpha (TGF-α), binds to the EGFR. This binding induces dimerization and autophosphorylation of the receptor on its intracellular tyrosine residues.

2. **GRB2 and SOS Recruitment**: The phosphorylated tyrosines on EGFR serve as docking sites for adaptor proteins, such as Growth factor receptor-bound protein 2 (GRB2). GRB2 is associated with Son of Sevenless (SOS), a guanine nucleotide exchange factor (GEF).

3. **RAS Activation**: SOS activates RAS, a small GTPase, by facilitating the exchange of GDP for GTP on RAS, thereby converting it from an inactive to an active state.

4. **RAF Activation**: Active RAS recruits and activates RAF kinase (which has three isoforms: A-RAF, B-RAF, and C-RAF/Raf-1) to the membrane.

5. **MEK Activation**: RAF phosphorylates and activates MEK1 and MEK2 (MAPK/ERK kinase), dual-specificity kinases that phosphorylate both threonine and tyrosine residues.

6. **ERK Activation**: MEK phosphorylates and activates ERK1 and ERK2. Once activated, ERK can translocate to the nucleus.

7. **Nuclear Translocation and Target Gene Expression**: In the nucleus, ERK phosphorylates various transcription factors, such as ELK1, which then bind to DNA and initiate the transcription of genes involved in cellular proliferation, differentiation, and survival.

The EGFR-ERK pathway is tightly regulated at various steps to ensure proper cellular responses. Dysregulation of this pathway is associated with various pathologies, including cancer, where mutations in components of the pathway can lead to uncontrolled cell growth and proliferation. Targeting components of the EGFR-ERK pathway with inhibitors is a therapeutic strategy in several cancers.
